# Understanding the biogeochemical mechanisms of metal removal from acid mine drainage with a subsurface limestone bed at the Motokura Mine, Japan

**DOI:** 10.1038/s41598-020-78069-9

**Published:** 2020-12-01

**Authors:** Shigeshi Fuchida, Kohei Suzuki, Tatsuya Kato, Masakazu Kadokura, Chiharu Tokoro

**Affiliations:** 1 Faculty of Science and Engineering, Waseda University, 3-4-1 Okubo, Shinjuku-ku, Tokyo, 169-8555 Japan; 2 Graduate School of Creative Science and Engineering, Waseda University, 3-4-1 Okubo, Shinjuku-ku, Tokyo, 169-8555 Japan

**Keywords:** Pollution remediation, Geochemistry

## Abstract

Subsurface limestone beds (SLBs) are used as a passive treatment technique to remove toxic metals from acid mine drainage (AMD). In this study, we investigated the mechanisms and thermodynamics of metal (manganese, copper, zinc, cadmium, and lead) precipitation in the SLB installed at the Motokura Mine. Field surveys in 2017 and 2018 showed that the pH of the SLB influent (initially 5–6) increased to approximately 8 in the drain between 24 and 45 m from the inlet. This increase was caused by limestone dissolution and resulted in the precipitation of hydroxides and/or carbonates of copper, zinc, and lead, as expected from theoretical calculations. Manganese and cadmium were removed within a pH range of approximately 7–8, which was lower than the pH at which they normally precipitate as hydroxides (pH 9–10). X-ray absorption near-edge structure analysis of the sediment indicated that δ-MnO_2_, which has a high cation-exchange capacity, was the predominant tetravalent manganese compound in the SLB rather than trivalent compound (MnOOH). Biological analysis indicates that microorganism activity of the manganese-oxidizing bacteria in the SLB provided an opportunity for δ-MnO_2_ formation, after which cadmium was removed by surface complexation with MnO_2_ (≡ MnOH^0^ + Cd^2+^  ⇄  ≡ MnOCd^+^  +  H^+^). These findings show that biological agents contributed to the precipitation of manganese and cadmium in the SLB, and suggest that their utilization could enhance the removal performance of the SLB.

## Introduction

Acid mine drainage (AMD) that contains large amounts of toxic metals, particularly manganese (Mn), iron (Fe), copper (Cu), zinc (Zn), arsenic (As), cadmium (Cd), and lead (Pb), is hazardous to the environment around active and abandoned mines. The addition of neutralizing agents, such as slaked and quick limes that form hydroxides, carbonates, surface complexation, and/or coprecipitation with ferrihydrite (Fe(OH)_3_) and gibbsite (Al(OH)_3_)^1–3^, is used to treat AMD with a high loading of toxic metals, especially Mn, Fe, and aluminum (Al). Passive treatments that use various natural geochemical and biological processes are also used as treatments instead of active treatments. Passive techniques require a certain maintenance cost but little resource input compared with active treatments using neutralizing agents^4,5^. Open limestone channels (OLCs) and limestone leach beds (LLBs) are the simplest construction and operation of the passive treatment systems that use large limestones. The OLCs tend to be constructed in a steep slope area and AMD is neutralized as it passes through the slope. LLBs are small basins and AMD is discharged with upward or downward flow after reaction in the basin^5,6^. These systems require a simple operation and low cost; however, the neutralizing capacity of the OLCs sometimes decreases significantly within a short period; this is because oxic neutralization of high-load AMD causes rapid clogging and coating of the limestone surfaces by hydroxides and carbonates of Fe and Al^5–7^. Wetlands are also used to remove metals by biogeochemical reactions involving vegetation, microorganisms, and organic matter in peat moss, spent mushroom compost, sawdust, and straw/manure^5,6,8^. To treat AMD of a high acidity, limestone bed agents are added to the system to supply bicarbonate ions continuously^6^. Limestone bed agents are a simple passive treatment system but it may be difficult to remove Mn from AMD that contains a high amount of Fe; this is because the Mn oxidation rate is slower than Fe and it inhibits or reverses the Mn oxidation^6,9^. Thus, these systems require enough area and residence time for the successful removal of Mn^5,6^.

Subsurface flow systems are developed as a technique to prevent contact of AMD with atmospheric O_2_. Buried trenches or beds are filled with limestone through which AMD is passed without exposure to the atmosphere and the escape of carbonate dioxide, which results in the promotion of limestone dissolution and an increase in AMD pH and alkalinity^5,6,10^. Anoxic limestone drains (ALDs), through which deoxygenated AMD flows, have been installed to treat AMD at many mine sites in the USA^11,12^ and Europe. Although Fe is not precipitated until pH 8 under deoxygenated conditions, excess dissolved Fe, Al, and O_2_ reduces the treatment performance and lifetime of the ALDs by clogging with their precipitants^6^. Thus, general guidelines recommend AMD introduction with elemental concentrations of below 1 mg L^−1^ into the ALDs to ensure effective operation^13,14^. Skousen and Ziemkiewicz described the performance of 36 ALDs that were installed in the USA. Most systems with a proposed lifetime of 20 years have been operating for more than the expected lifetime, although some systems lost their neutralizing ability after 8–9 years of operation^15^. The residence time of the ALD systems have shown that the highest removal efficiency was 2–6 h, whereas most systems had residence times between 20 and 100 h^15^. Hedin et al. developed an AMD sizing model based on the capacities of the ALDs installed at 13 coal mines in Pennsylvania, USA^13^. In their model, the minimum ALD size (m^2^) was determined by dividing the acidity loading (g day^−1^) by 7. This model was based on the difference in chemical composition before and after passage through the ALD but did not consider the specific biochemical reactions that occurred within the ALD^14^. In practice, pH ranges within which each metal is removed by precipitation and adsorption reactions differ depending on the water chemistry. To ensure that passive treatment systems treat AMD efficiently, the process must be scientifically tailored to the water quality and quantity at each mine. Thus, an increased understanding of the specific removal mechanisms and the development of geochemical models, for the different passive treatment options, is essential to choose the sizing processes that are best suited to the water quality and quantity at a particular mine.

A pilot-scale subsurface limestone bed (SLB) has been installed at the Motokura Mine (Hokkaido, Japan) since 2015. Although the SLB removes multiple toxic metals, the biogeochemical mechanisms by which it does so are unclear. In this study, we monitored changes in the drainage and sediment chemistries within the SLB and examined the specific removal mechanisms based on a chemical and biological analysis of the samples and chemical equilibrium calculations.

## Materials and methods

### Site description

**Figure 1:**
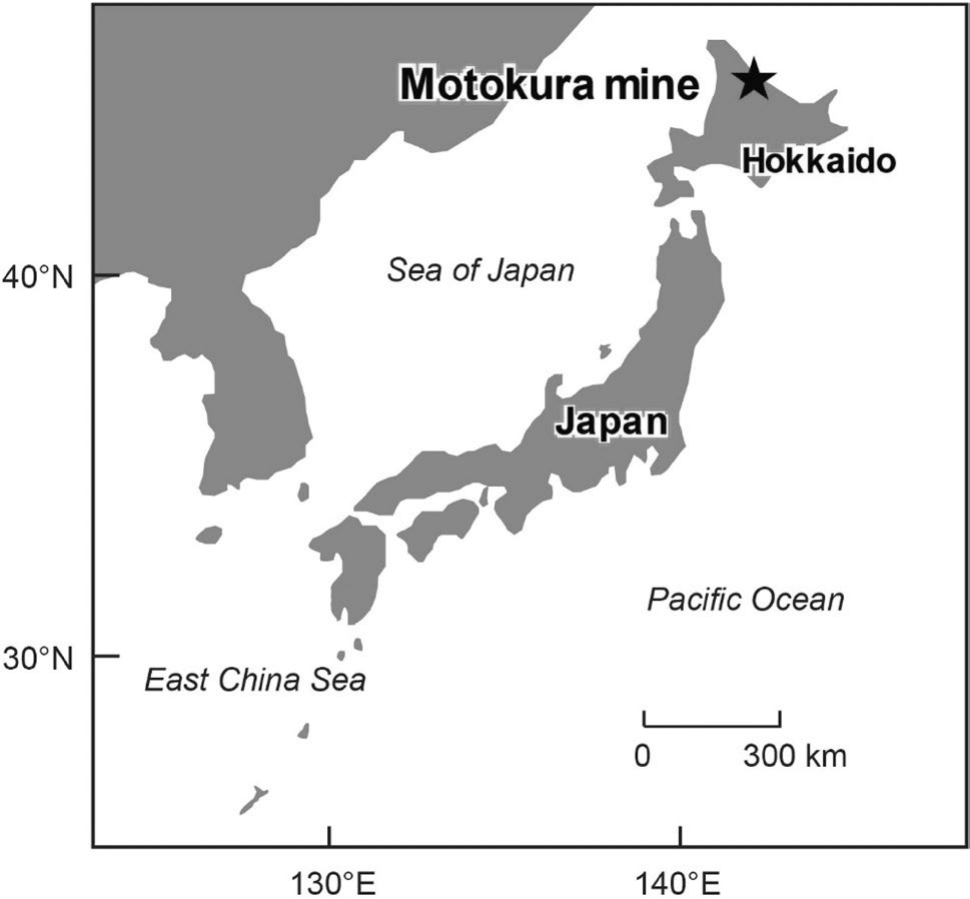
Location of the Motokura Mine in Hokkaido, Japan.

The Motokura Mine is located 23 km southwest of the mouth of the Tokushibetsu River, upstream of a tributary of the Ohun Tarmanai River in Hokkaido, Japan (Fig. 1). The Hokkaido government has been treating AMD at this mine since 1982. AMD that is discharged from the tunnels at the Motokura Mine contained 0.76–12 mg L^−1^ Fe, < 0.01–0.25 mg L^−1^ Cu, 0.090–0.52 mg L^−1^ Pb, 0.62–3.1 mg L^−1^ Zn, < 0.005–0.011 mg L^−1^ Cd, and < 0.005–0.28 mg L^−1^. The average AMD pH was 4.8–6.8, as measured between 2012 and 2013^16^.

**Figure 2:**
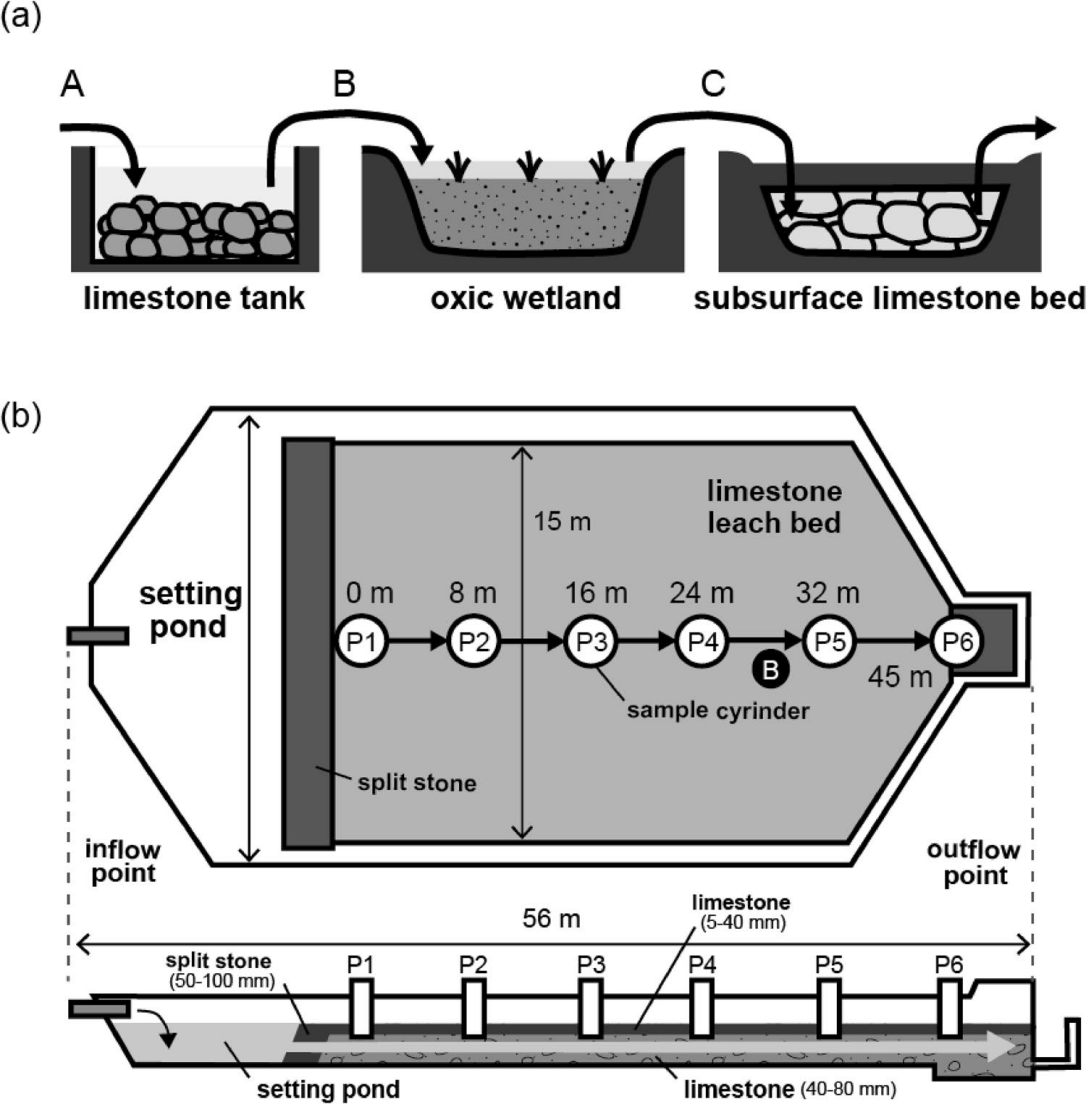
Schematic diagrams of (**a**) passive treatment system overview and (**b**) floor and cross-section plans of SLB installed at the Motokura Mine.

Three passive treatment systems (a limestone tank, an oxic wetland, and a SLB) were installed at the Motokura Mine in 2013^16^. The discharge from six sites (100 m, 70 m, Monju, Sanwa, Heiantsudo tunnels, and the deposit sites) is collected in the catchment basin, from which it flows in series into the limestone tank, oxic wetland, and SLB (Fig. 2a). We collected and monitored the quality and quantity of drainage that entered each system at two field surveys on May 25, 2017 and May 29, 2018. The chemical compositions of the drainage before/after entering each system are shown in Table 1.

**Table 1:** Chemical compositions of drainage before entrance into the limestone tank (raw water; Fig. 2a(A)), oxic wetland (outflow from limestone tank; Fig. 2a(B)), and SLB (outflow from oxic wetland; Fig. 2a(C)).

Point	pH	ORP (mV)	DO (mg dm^−3^)	EC (μS m^−1^)	Concentration (mg L^−1^)													
					Na	Mg	Ca	Al	Si	Mn	Fe	Cu	Zn	As	Cd	Pb	Cl	SO_4_
**2017**																		
Inflow to limestone tank (raw water)	4.6	460	8.7	22	7.1	3.4	17	1.0	24	0.23	0.25	0.39	4.1	0.0064	0.025	0.95	4.5	75
Inflow to oxic wetland (outflow from limestone tank)	4.2	490	8.6	24	7.3	3.5	18	1.1	24	0.24	0.25	0.42	4.3	0.0043	0.027	1.0	4.5	74
Inflow to SLB (outflow from oxic wetland)	5.0	300	7.5	11	7.3	3.6	16	0.87	21	0.33	0.055	0.37	3.8	0.0017	0.025	0.74	4.5	69
**2018**																		
Inflow to limestone tank (raw water)	5.5	210	10	20	6.9	2.5	18	0.29	43	0.23	2.9	0.17	2.2	0.016	0.0018	0.41	4.9	90
Inflow to oxic wetland (outflow from limestone tank)	5.3	220	9.6	20	7.1	2.6	21	0.29	44	0.23	2.5	0.17	2.2	0.010	0.0015	0.42	4.8	91
Inflow to SLB (outflow from oxic wetland)	4.7	320	7.5	20	7.0	2.7	20	0.48	33	0.28	0.092	0.19	2.2	< 0.001	0.0020	0.43	4.8	83

The limestone tank is used for AMD neutralization to pH 5–6 before flow into the oxic wetland. The pH and Ca concentrations did not change after the AMD passed through the limestone tank (Table 1), possibly because of a coating of the reactive limestone surface by Fe(OH)_3_, which was observed before the survey in 2017^16^. The oxic wetland was constructed to remove metals from the drainage using microorganism activities, such as those of iron-oxidizing and sulfur-reducing bacteria, fixation in vegetation, and adsorption by organic matter^16^. Our field surveys revealed that the concentrations of Fe and As decreased significantly after the AMD passed through the wetland, whereas other toxic metals (Mn, Cu, Zn, Cd, and Pb) were seldom removed (Table 1). This removal of Fe and As was caused because the oxidation of Fe^2+^ in the neutralized drainage was promoted by bioactivity and atmospheric air, after which As was coprecipitated by ferrihydrite formation^15^. The suspended solids that were produced by the oxidation of Fe and other metals could be removed by adsorption onto the reed vegetation and thus be immobilized in the wetland. The SLB was installed to remove residual toxic metals (Mn, Cu, Zn, Cd, and Pb) in the drainage after passing through the oxic wetland. The upper portion of the channel (~ 40 cm) contained limestone pebbles that were less than 40 mm in diameter, whereas the lower part (40–120 cm) of the structure contained limestone gravel with a particle size of 40–80 mm. Drainage that entered the SLB from the wetland flowed through the gaps in the calcareous gravel (Fig. S1). The average inflow rates were 131–142 L min^−1^ but the outflow rates were 60.6–70.8 L min^−1^, and it is estimated that approximately half the drainage volume leaked during its passage through the SLB.

### Sample collection from SLB

Water and sediment samples from the SLB were collected during two field surveys on May 25, 2017 and May 29, 2018. Water samples were collected using cylinders installed at points P1–P6 in the SLB (Fig. 2b). The pH, electrical conductivity (EC), dissolved oxygen (DO), and oxidation reduction potential (ORP) of the water samples were measured during sample collection. The water sample was filtered through a 0.1-μm mixed cellulose ester membrane filter (ADVANTEC Japan Co. Ltd., Japan, Tokyo) and preserved with HNO_3_ (1%) in a polypropylene bottle for subsequent determination of the cation and anion compositions. Sediment samples of the calcareous gravel were taken at a depth of 50 cm from the surface at point B in the SLB, and preserved in a freezer in the dark for biological and chemical analysis to avoid oxidation and other chemical alteration of the samples.

### Quantification of chemical components in water samples

The concentrations of sodium (Na), magnesium (Mg), calcium (Ca), Al, Mn, Fe, Cu, Zn, As, Cd, and Pb in the filtrates were quantified using inductively coupled plasma-mass spectrometry (ICP-MS, Agilent, 7700X, Agilent Technology, US, CA). The chlorine (Cl) and sulfate (SO_4_) concentrations in the filtrates were determined by ion chromatography (IC, ICS-2100, Thermo Fisher Scientific Inc, US, MA) in our laboratory.

### XAFS analysis

X-ray absorption fine structure (XAFS) analysis at the Mn K-edge was performed using the BL5S1 beamline at Aichi Synchrotron Radiation Center, Japan, and the BL14B2 beamline at the SPring-8 Synchrotron Radiation Facility, Japan. This analysis can measure low concentrations of amorphous compounds, such as biogenic minerals, which is difficult to do by other non-destructive techniques (such as X-ray diffraction and X-ray fluorescence). The sediment sample was freeze-dried, diluted with boron nitride, and formed into pellets of 10 mm diameter and 1.0 mm thickness. All XAFS spectra at the Mn K-edge were collected over the energy range 6200–7200 eV by the transmission method using silica (111) monochromator crystals. Birnessite (δ-MnO_2_) and manganite (γ-MnOOH) were used as reference materials. After normalizing for the edge jump, X-ray absorption near-edge structure (XANES) analysis was conducted in the energy range 6540–6590 eV to identify manganese mineral species. The XANES analyses were performed using the Athena software package^17^.

### Geochemical modeling using PHREEQC

Geochemical code PHREEQC ver.2 (USGS) was used to simulate the behavior of each element on its path through the SLB by coupling various chemical reactions^18^ (i.e., precipitation, surface complexation, iron and manganese oxidation, and calcite dissolution) and one-dimensional advection analysis. The specific chemical species, surface complexation reactions, and equilibrium constants were published in a previous study^19^. The adsorption equilibrium constant of Cd onto δ-MnO_2_ (≡ MnOH^0^ + Cd^2+^  ⇄  ≡ MnOCd^+^  + H^+^; Log *K* = 2.9) was obtained by an adsorption experiment (details of this procedure are described in the supporting information). The equations for Mn oxidation and calcite dissolution reaction rates were given by Singer and Stumm^20^ and Plummer et al.^21^, respectively, and are explained in the supporting information).

### Other software

Output data and measured values were summarized and schematic diagrams were produced using Microsoft Excel, and diagrams were drawn using Adobe Illustrator.

## Results and discussion

### Change in drainage quality during flow through SLB

**Figure 3:**
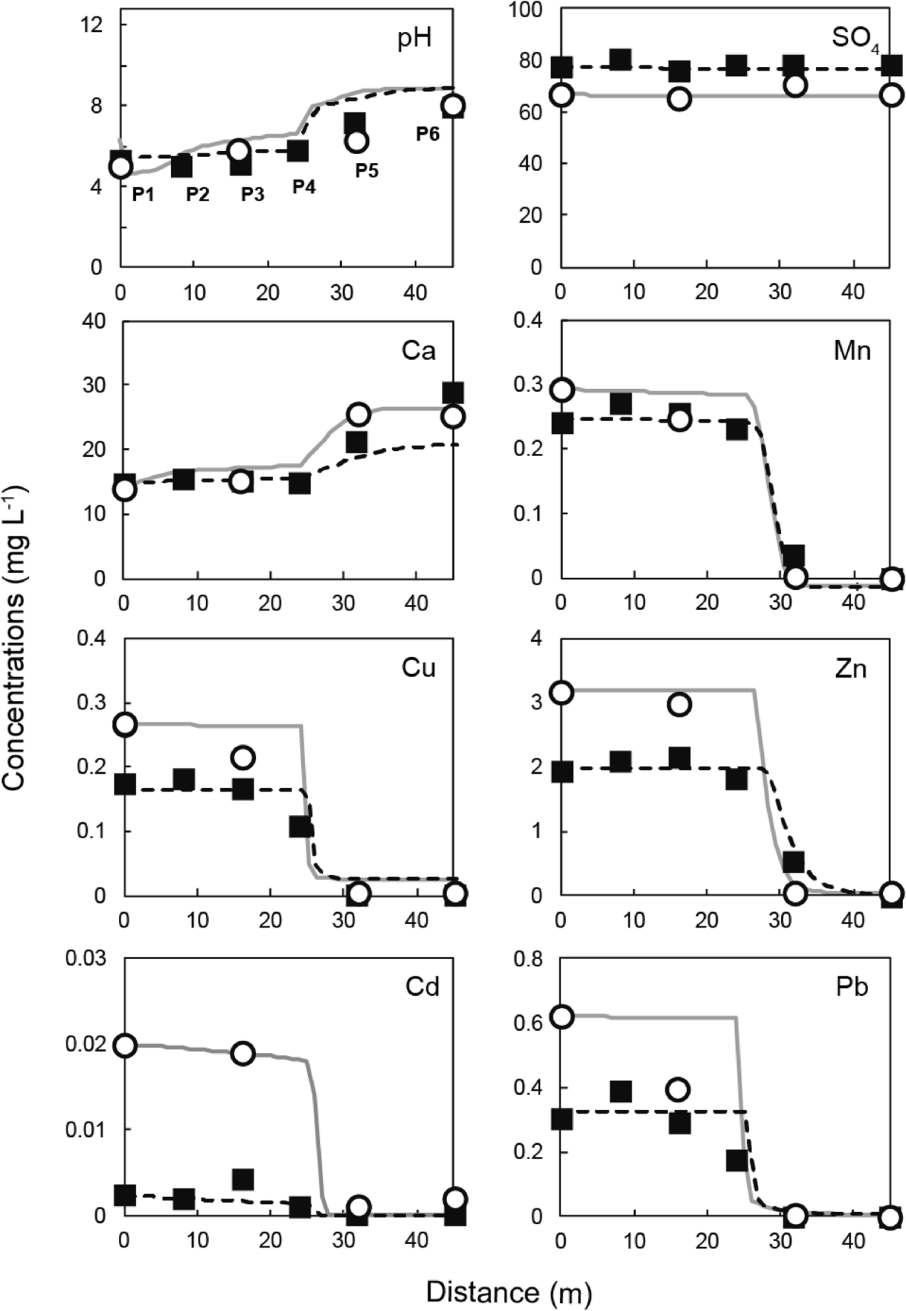
Changes in measured pH values and concentrations of each element of the drainage during flow through the SLB. The measured values of the water samples in 2017 are shown as open circles, whereas measurements from 2018 are shown as black squares. The lines are simulated values obtained using PHREEQC.

Significant changes in the drainage quality during flow through the SLB were observed in two field surveys. The measured pH values and concentrations of each element are shown in Table 2 and Fig. 3. The pH of the drainage that was introduced into the SLB (5–6) changed little until 24 m into the SLB. Between 24 and 45 m, the pH increased to approximately 8, and the Ca concentration increased from 14–16 to 21–29 mg L^−1^. This increase occurred because limestone dissolution began approximately 24 m into the SLB. XRD analysis revealed that calcite was dominant in the limestone, which caused Ca concentration patterns that are indicative of neutralization by limestone. As the pH increased, the concentration of all metals (Mn, Cu, Zn, Cd, and Pb) decreased below the analytical quantifiable limits at the end of the SLB. These metals were precipitated as secondary minerals by neutralization reactions. The DO and ORP values were measured in the cylinders at various points in the SLB (Table 2(a)). The DO remained in the range of 5–9 mg L^−1^ over the entire SLB, possibly because samples in the cylinders were in equilibrium with the atmosphere. The ORP values decreased slightly from 396 to 211 in 2017 and 331 to 268 mV in 2018 over the length of the SLB. The high DO and ORP values indicate that drainage that was introduced to the SLB did not become deoxygenated, and the decrease in ORP indicates oxygen consumption by the removal of metal ions and other geochemical reactions, including microbial activity.

**Table 2:** Chemical composition of drainage collected from points in the SLB in 2017 and 2018.

(a)														
Point	Distance (m)	pH	ORP (mV)	DO (mg L^−1^)	EC (μS m^−1^)									
**2017**														
P1	0	5.1	396	7.8	17									
P3	16	5.8	414	7.9	17									
P5	32	6.3	392	8.1	20									
P6	45	8.1	211	7.2	20									
**2018**														
P1	0	5.4	331	8.4	18									
P2	8	5.0	323	7.0	19									
P3	16	5.2	289	6.6	17									
P4	24	5.8	281	7.6	18									
P5	32	7.2	245	5.2	19									
P6	45	8.0	268	8.7	26									

(b)														
Point	Concentration (mg L^-1^)													
	Na	K	Mg	Ca	Al	Si	Mn	Fe	Cu	Zn	Cd	Pb	Cl	SO_4_
**2017**														
P1	7.1	0.74	5.2	14	1.58	21	0.29	0.037	0.27	3.2	0.020	0.62	4.4	68
P3	6.8	0.70	5.1	15	0.43	19	0.25	0.015	0.21	3.0	0.019	0.39	4.4	66
P5	6.6	0.85	4.5	26	0.017	13	0.0036	0.011	0.0021	0.042	0.0011	0.0020	4.4	71
P6	6.6	0.81	4.7	26	0.030	15	0.0002	0.0031	0.0019	0.049	0.0019	0.0014	4.4	68
**2018**														
P1	6.3	1.2	2.8	15	0.23	3.2	0.25	< 0.0001	0.17	2.0	0.0024	0.31	4.7	78
P2	6.6	1.2	2.7	16	0.36	3.4	0.27	0.026	0.18	2.1	0.0018	0.39	4.8	81
P3	6.1	1.1	2.8	15	0.31	3.2	0.26	< 0.0001	0.16	2.2	0.0042	0.29	4.7	76
P4	6.4	1.2	2.8	15	0.14	3.2	0.23	< 0.0001	0.11	1.8	0.0011	0.17	4.8	79
P5	5.5	1.9	3.3	21	0.0073	1.7	0.037	< 0.0001	< 0.0001	0.56	< 0.0001	< 0.0001	4.9	79
P6	6.2	1.1	3.4	29	0.0031	2.1	< 0.0001	< 0.0001	< 0.0001	< 0.0001	< 0.0001	< 0.0001	4.8	79

(a) Measured values of pH, oxidation–reduction potential (ORP), dissolved oxygen (DO), and electric conductivity (EC) at the SLB. (b) concentrations of major elements.

The pH values at the decreasing metal concentrations were 6.3–7.2 (P4–P5 in Fig. 3). Hydroxides of Cu and Pb were calculated to precipitate at a pH close to 6, which agreed with the field observations in the SLB. A higher pH was generally required to remove Mn, Zn, and Cd by hydroxide formation. Mn (II) is oxidized to Mn (III) at a pH of approximately 9, and precipitates as manganese oxyhydroxide (MnOOH)^22^, and Zn and Cd precipitate at pH values of approximately 8 and 9–10, respectively^23^. However, the concentration of Zn, Mn, and Cd decreased at a pH of approximately 7–8, which is lower than the pH at which their hydroxides normally precipitate. This behavior suggests that other factors, such as bioactivity and water chemistry, along with neutralization, contribute substantially to the removal of Mn and Cd in the SLB.

### Characteristics of manganese oxide in the sediment sample of SLB

**Figure 4:**
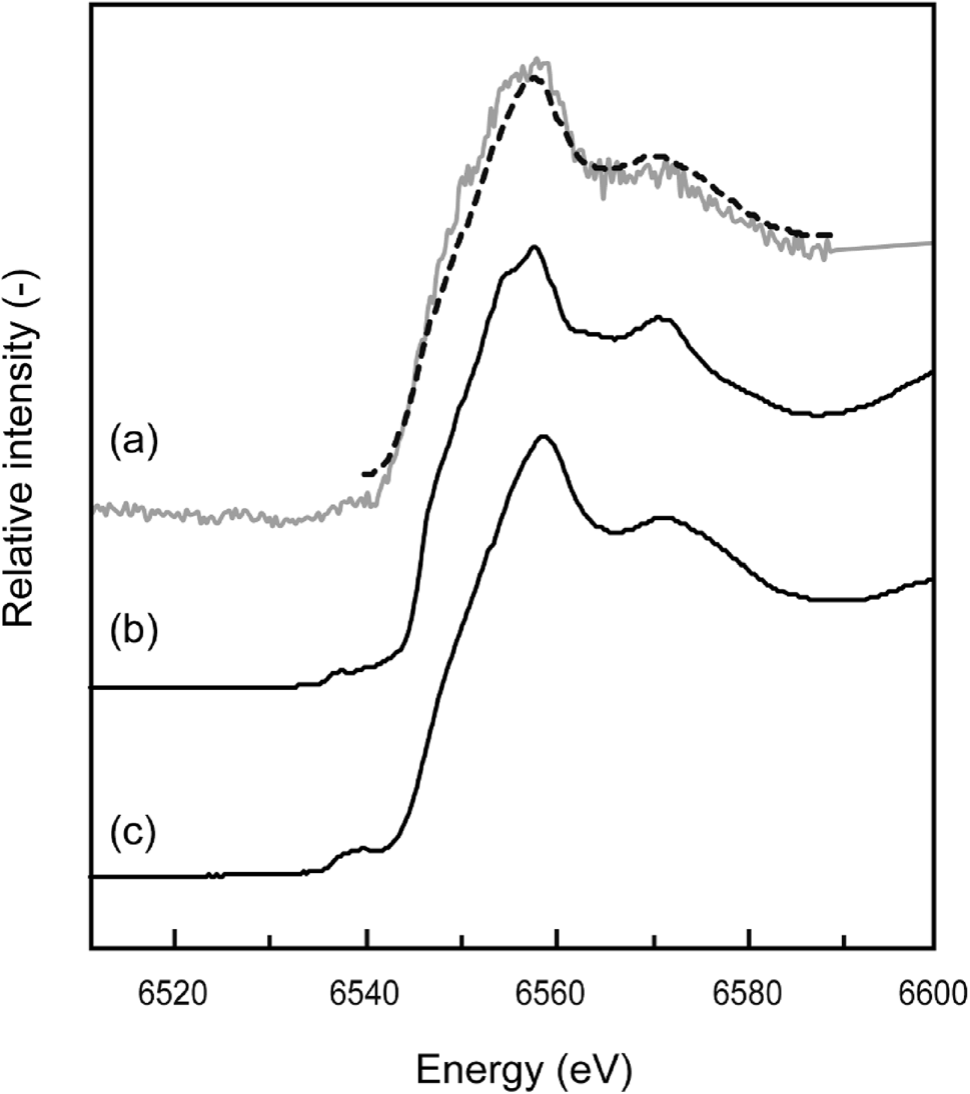
Mn-edge XANES spectra of (**a**) sediment samples and reference materials (**b**) MnOOH and (**c**) δ-MnO_2_. The solid and broken lines indicate the measured and fitted results, respectively.

The mineralogy and chemistry of manganese oxide collected from point B of the SLB (shown in Fig. 2a) were analyzed to identify the specific removal mechanism of Mn and Cd from the neutral-pH drainage. Different manganese oxides formed depending on the oxidation state of Mn: γ-MnOOH for Mn (III) and δ-MnO_2_ for (IV). The type of Mn oxide was determined from the oxidation state obtained by Mn-edge XAFS analysis. The fit of the sample XANES spectra to the reference materials showed that δ-MnO_2_ was predominantly formed (> 99%) (Fig. 4). In general, the oxidation of Mn(II) to Mn(III) and M(IV) is slow in an abiotic system, with a half-life of a few hundred days or years even under alkaline condition (pH of 8–10 at 25 °C), and the main products are MnOOH (manganite) or other Mn(II)/(III) mixture compounds^22,24^. The MnOOH is thermodynamically metastable and gradually transforms to MnO_2_, but this reaction depends on ill-defined physicochemical conditions, and often takes months or years^25^. Thus, theoretically, manganite and other low-oxidation-state compounds, such as Mn_3_O_4_, should have been found in the SLB when abiotic oxidation was dominant. However, our XAFS analysis showed clearly that the Mn(II) in the AMD was oxidized to Mn(IV), which resulted in the formation of δ-MnO_2_ in the SLB.

This rapid oxidation of Mn could be attributed to the presence of Mn-oxidizing bacteria in the SLB. Real-time PCR analysis of the 16S rDNA gene of eubacteria collected from the sediment samples indicated the existence of two types of Mn-oxidizing bacteria: *Pseudomonas* and *Bosea*^26^. Villalobos et al. reported that *Pseudomonas* species can produce a layered Mn oxide that is close in structure to δ-MnO_2_^27^. Furuta et al. explained that MnO_2_, with an amorphous and fibrous structure from Mn (II) (over 5.5 mg L^−1^), can be synthesized by the activity of *Bosea* species at neutral pH (6.0–6.3) and under anaerobic (approximately 5–10% oxygen in the gas phase) conditions^28^. Once δ-MnO_2_ is formed, Mn oxidation is promoted by autocatalytic reaction with adsorption of Mn^2+^ on the δ-MnO_2_ surface. Therefore, δ-MnO_2_ would be formed initially by the activity of Mn-oxidizing bacteria and gradually accumulated by the combination of microbial and autocatalytic oxidation in the SLB. The ion-exchange capacity of δ-MnO_2_ (240 meq/100 g) is higher than that of other metal oxides and even clay minerals, such as montmorillonite^29^. Recent studies have revealed that δ-MnO_2_ can adsorb Cd from solution at pH 6 by surface complexation and coprecipitation^30^. They explained that the removal mechanisms can change depending on the Cd/Mn molar ratio; surface precipitation and/or intercalation can occur when the Cd/Mn molar ratio exceeds 1, whereas simple surface complexation becomes the dominant reaction at a lower Cd/Mn molar ratio (< 1). On the basis of these reports, the Cd/Mn molar ratios in the drainage that were installed in the SLB were low (0.033 in 2017 and 0.0049 in 2018), which indicates that Cd could be removed mainly by surface complexation formation on δ-MnO_2_ in the SLB. The δ-MnO_2_, which has a high affinity for metal cations, was most likely formed by Mn-oxidizing bacteria and not by abiotic oxidation, and this plays an important role in efficient removal of Cd from the drainage even at neutral pH.

### Geochemical modeling of SLB in the Motokura Mine

Changes in the chemical composition of the flow through the SLB were simulated using equilibrium calculations in PHREEQC, including precipitation, surface complexation, Fe and Mn oxidation, and calcite dissolution reactions. The simulation results compared well with the measured values from two field surveys (Fig. 3). The composition at point P1 was used as the initial composition for the calculations (Table 2), and the flux was calculated by dividing the flow rate in the SLB by its cross-sectional area. The actual flow rates in 2017 and 2018 were 0.042 and 0.052 mL min^−1^, respectively. The parameters for each kinetic calculation were determined by fitting to the measured values. For the Mn oxidation rate, the calculated rate constants *k*_1_ and *k*_2_ (see Eq. 2 in the supporting information) were 4.9 × 10^–7^ and 2.1 × 10^10^, respectively. The Mn-oxidation rate in the SLB caused by the activity of Mn-oxidizing bacteria was significantly faster than the oxidation caused by atmospheric air. Different microbial species and densities may be present at other field locations. The rate constants for calcite dissolution, *k*_1_, *k*_2_, and *k*_3_, (see Eq. 3 in the supporting information) were those reported by Plummer et al.^21^, and the reactive surface area of calcite exposed to water was calculated by fitting. The exposed calcite reactive surface area decreased gradually along the length of the SLB because of secondary mineral formation caused by the neutralization. Different Ca concentration patterns were found in the upstream (0–24 m) and downstream (25–45 m) portions of the SLB. These regions were treated separately in the calculations (Table 3). The reactive surface area of calcite was 23–33 times smaller in the upstream portion than in the downstream portion, which suggests that the neutralization capacity of the calcite in the upstream portion was diminished by reaction with the drainage. Surface complexation that led to Cd removal in the SLB was controlled by adsorption on the δ-MnO_2_ surface as explained above. No adsorption equilibrium constant for this reaction has been reported in the literature to the best of our knowledge. We therefore experimentally investigated the adsorption of Cd by δ-MnO_2_ and found the value of the adsorption equilibrium constant to be Log *K* = 2.9 (see supporting information for details) (Fig. S3). As shown in Fig. 3, the calculated values for pH and each metal corresponded well with the values measured in the two field surveys.

**Table 3:** Reactive surface area of limestone in contact with water, estimated by fitting the chemical analyses of solution samples.

Year	Reactive surface area (m^2^ L^−1^)	
	Upstream (0–24 m)	Downstream (25–45 m)
2017	0.0008	0.026
2018	0.0006	0.014

**Figure 5:**
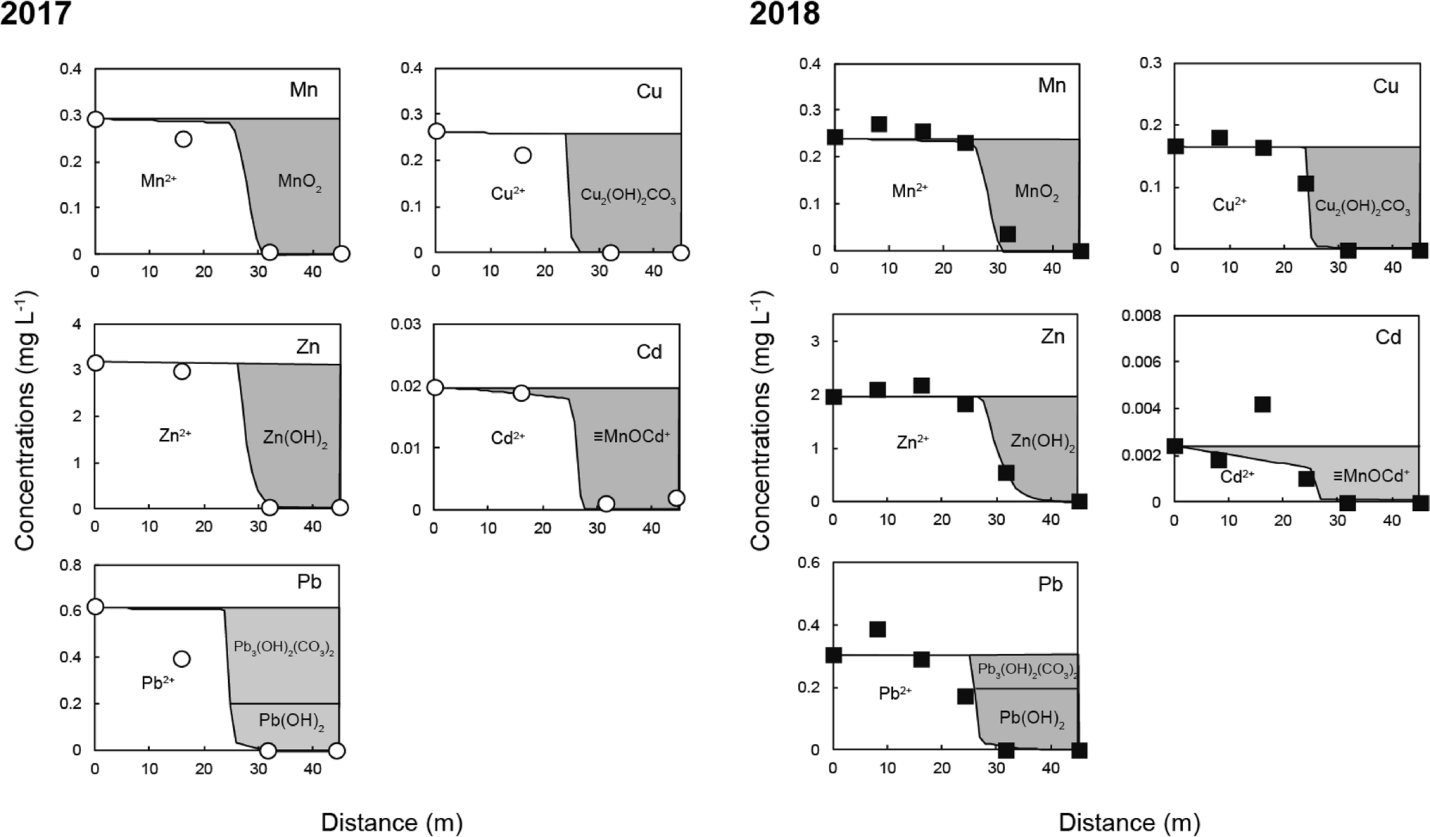
Changes in dissolved Mn, Cu, Zn, Cd, and Pb species during flow through the SLB.

The changes in dissolved Mn, Cu, Zn, Cd, and Pb during flow through the SLB are shown in Fig. 5. The Zn and most of the Pb (> 60%) precipitated as hydroxides as the pH in the SLB increased. The Cu and some of the remaining Pb formed carbonates Cu_2_(OH)_2_CO_3_ (malachite) and Pb_3_(OH)_2_(CO_3_)_2_ (hydrocerrusite), respectively. Cd was removed at neutral pH by surface complex formation on MnO_2_ by microbial activity. The adsorption equilibrium constant for the surface complexation reaction of Cd on MnO_2_ (Log *K* = 2.9) indicates that δ-MnO_2_ has a significantly greater Cd removal ability at neutral pH than ferrihydrite (Log *K* = 0.47), which is a widely used adsorbent for many toxic metals^31^. Therefore, the promotion of δ-MnO_2_ formation by Mn-oxidizing bacteria is an important factor to control Cd behavior in the SLB, and these results show that the behavior of metals in SLBs cannot be accounted for by abiotic chemical reactions alone.

As shown in Table 3, the estimated reactive surface area of calcite exposed to water was 23–33 times smaller in the upstream portion of the SLB than in the downstream portion. This estimate decreased slightly in 2018 compared with 2017. This means that the neutralization capacity of the calcite was gradually reduced over time because of coating by secondary mineral formation in the SLB. The Ca concentrations in the upstream portion of the SLB were in the same range in 2018 and 2017 (1.4–1.6 mg L^−3^), which indicates that less calcite reacted with AMD. Many studies have investigated the coating of limestone surfaces by the formation of Fe(OH)_3_, Al(OH)_3_, and gypsum (CaSO_4_) by laboratory-scale experiments using calcite-paved columns^32,33^. Soler et al. studied how the neutralization capacity of calcite was reduced by coating. Artificial AMD that contained 250–1500 mg L^−1^ Fe and 250–1500 mg L^−1^ SO_4_ at pH 2 was passed through 1.2–6.0 cm columns packed with limestone particles of approximately 5 mm in size^33^. The reactive surface area of limestone decreased by half over 230 h, and after 300 h it was covered by Fe(OH)_3_ and CaSO_4_. In an SLB, the precipitation of metals (Zn, Mn, Cu, and Pb) and CaSO_4_ could reduce the neutralization capacity of limestone. Periodic dredging is therefore required to maintain the treatment capacity of an SLB. For the SLB in the Motokura Mine, the rate at which the SLB loses its neutralization capacity has not yet been determined because the limestone in SLB was not dredged since its installation in 2013. The secondary mineral formation on calcite would provide a reason for the low calcite dissolution in the SLB for our field surveys in 2017 and 2018.

As explained above, the drainage that entered the SLB contained a high amount of DO over 7 mg L^−1^, whereas the general ALDs are commonly used to treat deoxygenated drainages (< 1 mg L^−1^) that contain low quantities of Fe and Al (< 1 mg L^−1^)^4,5^. In the ALD system, AMD can pass through without exposure to the atmosphere and escape of carbon dioxide, which results in the promotion of limestone dissolution and an increase in AMD pH and alkalinity. For the many cases of ALD in the USA, the AMD pH increased to ~ 6.5 after passing through the ALD, which is lower than that of the SLB (8.0–8.1)^15^. All metals were removed at a pH above 7 in the SLB, which implies that efficient metal removal from the drainage in the Motokura Mine could be difficult by using conventional ALDs. Watzlaf et al. reported changes in the chemical composition of AMD before and after contact with the ALD systems, and revealed that the concentrations of Mn, Co, Ni and Zn did not decrease in outflowing drainage, whereas the pH increased to ~ 6.5^10^. Thus, additional treatments are required to remove those metals from the drainage. For the Motokura Mine, oxic wetland that was constructed as a pretreatment for the SLB was estimated to contribute to enhancing the removal efficiency of Mn and Cd from the drainage in the SLB. Organic matter and other nutrients, such as nitrogen and phosphate, could be supplied to drainage in the oxic wetland, which resulted in a contribution of the activity of Mn-oxidizing bacteria in the SLB because this organism is generally heterotrophic^34^. Calculations based on our geochemical model show that at least 15.5–18.3 h of residence time was required to meet the effluent standards using an SLB with a volume of 670 m^3^. This system size was larger than the general ALD that was installed in the USA for treatment of AMD with the same chemical composition and flow rates as the Motokura Mine^15^. The pH change during flow through the SLB indicates that approximately half of the system volumes had lost their neutralization capacity after 5 years because of coating and clogging by metal secondary minerals, which would be shorter than the general ALD lifetime of 20 years. However, the SLB system achieves the efficient removal of metals that co-present in the drainage, and especially aerobic organisms and their products can enhance the removal of Mn and Cd. The rate at which the SLB loses its neutralization capacity may vary depending on the quality and quantity of the AMD, and thus, methods to calculate the capacity should be developed in the future.

## Conclusions

The removal of metals (Mn, Cu, Zn, Cd, and Pb) by an SLB that was installed at the Motokura Mine was evaluated by field analysis and chemical equilibrium calculations. The pH of drainages that were introduced to the SLB (initially 5–6) increased to approximately 8 between 24 and 45 m from the SLB inlet. This was caused by limestone dissolution, and was followed by the precipitation of Cu, Zn, and Pb as hydroxides and/or carbonates. The experimental results matched the thermodynamic calculations well. Mn and Cd were removed in a pH range of approximately 7–8, which is lower than the pH at which they normally precipitate as hydroxides (pH 9–10). The removal occurred because the formation of δ-MnO_2_ was accelerated in the SLB and surface complexation of Cd occurred with its surface. Chemical and biological analysis of the samples and adsorption experiments confirmed the results. Therefore, Mn-oxidizing bacteria and δ-MnO_2_ production contribute to the efficient removal of Mn and Cd at neutral pH without excessive alkalization in a pilot-scale SLB. The chemical equilibrium calculations, which include the geobiochemical reactions, indicate that 15.5–18.3 h of residence time is required to meet Japanese effluent standards for the 670-m^3^ SLB at the Motokura Mine. Geochemical mechanisms and calculations, such as in this work, will be useful for determining a priori whether to install SLBs in mines with different quantities and qualities of AMD.

## Supplementary information

Supplementary Information.
