# Supplementary material for: Understanding the biogeochemical mechanisms of metal removal from acid mine drainage with a subsurface limestone bed at the Motokura Mine, Japan
Source: Sci Rep. 2020 Dec 1;10:20889. doi: 10.1038/s41598-020-78069-9 (PMC7708634; doi:10.1038/s41598-020-78069-9)
Supplement: Supplementary file 1 — Supplementary Information. [file 41598_2020_78069_MOESM1_ESM.docx]

**Supporting Information**

**Understanding the biogeochemical mechanisms of metal removal from acid mine drainage with a subsurface limestone bed at the Motokura Mine, Japan**

Shigeshi Fuchida^1^, Kohei Suzuki^2^, Tatsuya Kato^1^, Masakazu Kadokura^2^, Chiharu Tokoro^1*^

*^1^Faculty of Science and Engineering, Waseda University, 3-4-1 Okubo, Shinjuku-ku, Tokyo 169-8555, Japan*

*^2^Graduate School of Creative Science and Engineering, Waseda University, 3-4-1 Okubo, Shinjuku-ku, Tokyo 169-8555, Japan*

----------------------------------------------------------------------------

*Corresponding author: Chiharu Tokoro ([tokoro@waseda.jp](mailto:tokoro@waseda.jp))

TEL +81-3-5286-3320 FAX +81-3-5286-3491

**1. Photograph of subsurface limestone bed**


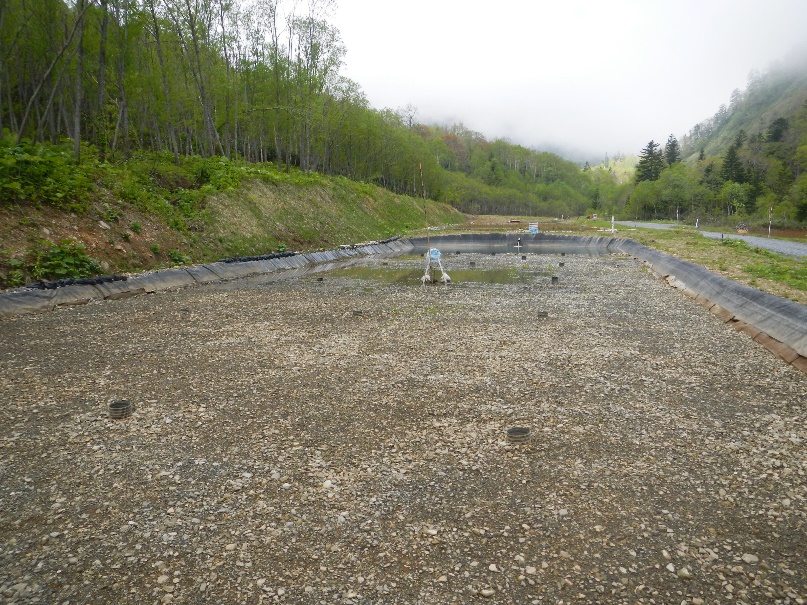


**Figure S1**. Photograph of subsurface limestone bed (SLB) installed at the Motokura Mine.

**2. Kinetics equations**

The equations for Mn oxidation and the calcite dissolution reaction rates were given by Singer and Stumm 1970^20^ and Plummer et al. 1978^21^, respectively.

The parameters for each kinetic calculation were determined by fitting the measured values from two field surveys.

For the Mn oxidation rate, the rate constants (*k*_1_ and *k*_2_) were calculated by fitting because the Mn oxidation rate from Mn-oxidizing bacteria was significantly faster than Mn oxidation by air in the ALD. Microbial species and densities may differ in different field locations.

| $\frac{\text{-}\text{d}\text{[Mn}\left( \text{III, IV} \right)\text{]}}{\text{dt}}\text{=}\frac{\text{d}\text{[Mn}\left( \text{II} \right)\text{]}}{\text{dt}},$ | (1) |
| --- | --- |
| $\frac{\text{-}\text{d}\text{[Mn}\left( \text{II} \right)\text{]}}{\text{dt}}\text{=}\left( \text{k}_{\text{1}}\text{+ }\text{k}_{\text{2}}\text{[OH}^{\text{-}}\text{]}^{\text{2}}\text{P}_{\text{O2}} \right)\text{[Mn}\left( \text{II} \right)\text{]}\text{,}$ | (2) |

where [Mn(III, IV)], [Mn(II)] and [OH-] are the activities of each chemical species and *P*_O2_ is the partial pressure of oxygen.

For calcite dissolution, the rate constants (*k*_1_ *k*_2_ and *k*_3_) used were those reported by Plummer et al. (1978)^21^ (defined in PHREEQC), and the surface area of calcite exposed to water was calculated by fitting. The exposed calcite surface area decreased gradually because of coating by secondary minerals that formed from neutralization.

| $\frac{\text{dC}_{\text{calcite}}}{\text{dt}}\text{=}\text{k}_{\text{f}}\text{S}\text{[1-}{\text{(}\frac{\text{IAP}}{\text{K}_{\text{calcite}}}\text{)}}^{\frac{\text{2}}{\text{3}}}\text{]}\text{,}$ | (3) |
| --- | --- |
| $\text{k}_{\text{f}}\text{= }\text{k}_{\text{1}}\left[ \text{H}^{\text{+}} \right]\text{+}\text{k}_{\text{2}}\left[ \text{CO}_{\text{2}} \right]\text{+}\text{k}_{\text{3}}\text{[}\text{H}_{\text{2}}\text{O]}\text{,}$ | (4) |

where *C*_calcite_ is the calcite concentration, *IAP* is the activity product of calcite (=[Ca^2+^] [CO_3_^2-^]), *K*_calcite_ is the solubility product of calcite, and *S* is the contact area between water and calcite.


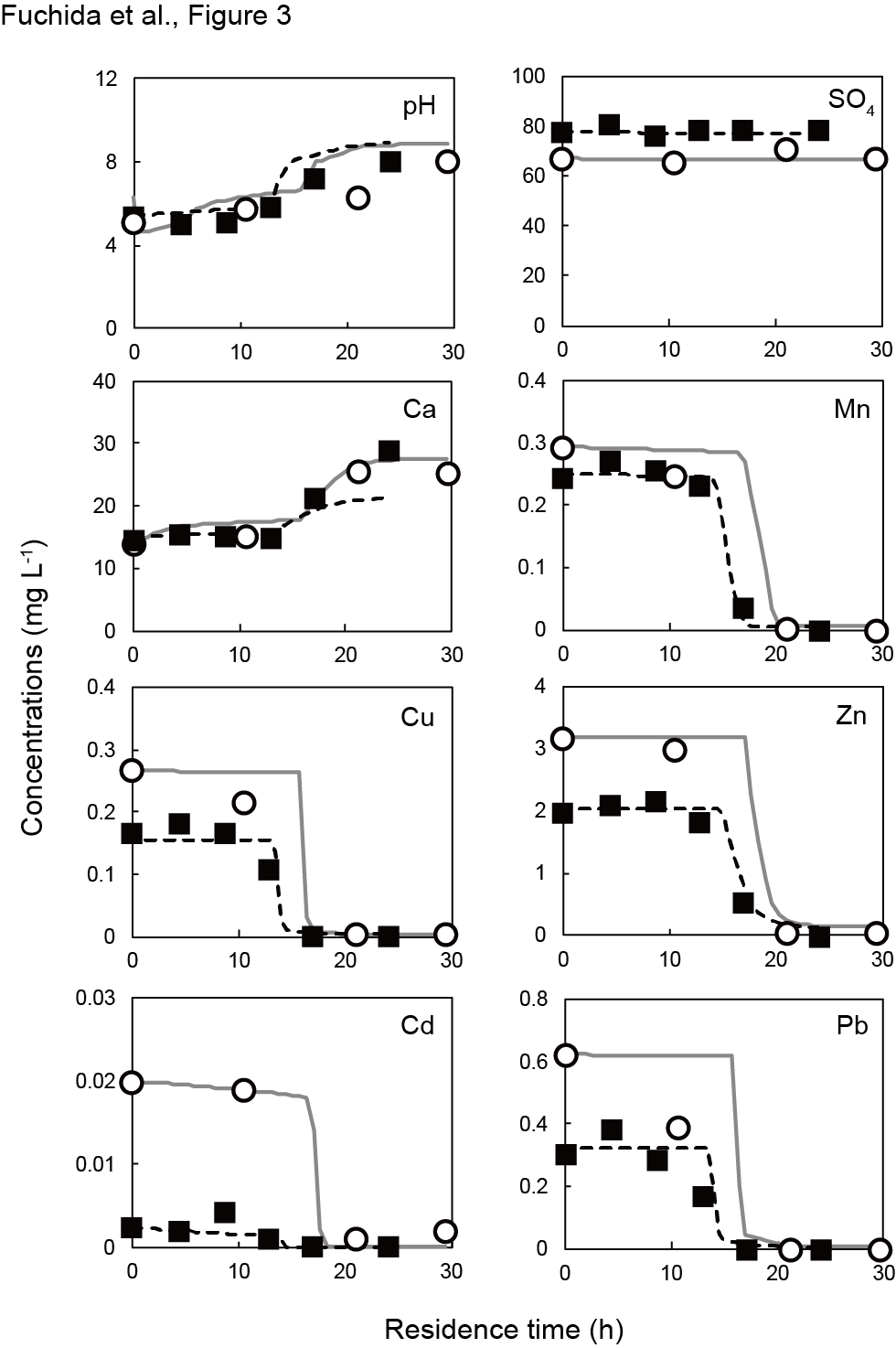
**3. Relationship between concentrations of each element versus residence time**

**Figure S2.** Changes in measured pH and concentrations of each element of drainage versus residence time during flow through the SLB. The measured values of water samples in 2017 are shown as open circles, whereas measurements from 2018 are shown as black squares. The lines are simulated values obtained using PHREEQC.

**4. Adsorption of Cd on MnO_2_**

For the thermodynamic simulation of Mn and Cd in the ALD, the adsorption equilibrium constant (*K*) of the surface complexation reaction of Cd with MnO_2_ (i.e., (≡MnOH^0^ + Cd^2+^ ⇄ ≡MnOCd^+^ + H^+^) was obtained from an adsorption experiment at pH 3.5–7.0. The MnO_2_ used in the experiment was synthesized as described in our recent study^28^. Suspensions of MnO_2_ (0.1 L) and Cd (0.1 L) were prepared in separate beakers. The initial concentrations of δ-MnO_2_ and Cd were doubled to account for dilution upon mixing (total volume: 0.2 L). The initial concentrations of Cd(II) were set to 0.266 and 0.356 mmol L^-1^, and Mn(II) solutions were prepared to obtain two different Cd/Mn molar ratios: 0.130 and 0.157. The solutions were mixed and stirred with a magnetic stirring bar for 1 h. The reactant was filtered through a 0.1-μm membrane filter (ADVANTEC, mixed cellulose ester), and the filtrate was preserved with 1 M HNO_3_ (1 wt%) in a polypropylene bottle for subsequent determination of Cd and Mn concentration by ICP-MS analysis.

The calculated ion-exchange capacity of the MnO_2_ used in the experiment was 0.23 mol mol-Mn^-1^ based on the surface area (89.8 m^2^ g^-1^) that was measured by using an Accelerated Surface Area and Porosimetry System (ASAP, Shimazu, Japan, Kyoto). The adsorption equilibrium constant for the surface complexation reaction of Cd with MnO_2_ (Log *K* = 2.9) was calculated by fitting to the experimental results using PHREEQC. For this calculation, the equilibrium constants for the desorption reaction of MnO_2_ and H^+^ given by Yao and Millero (1996) were used.

**
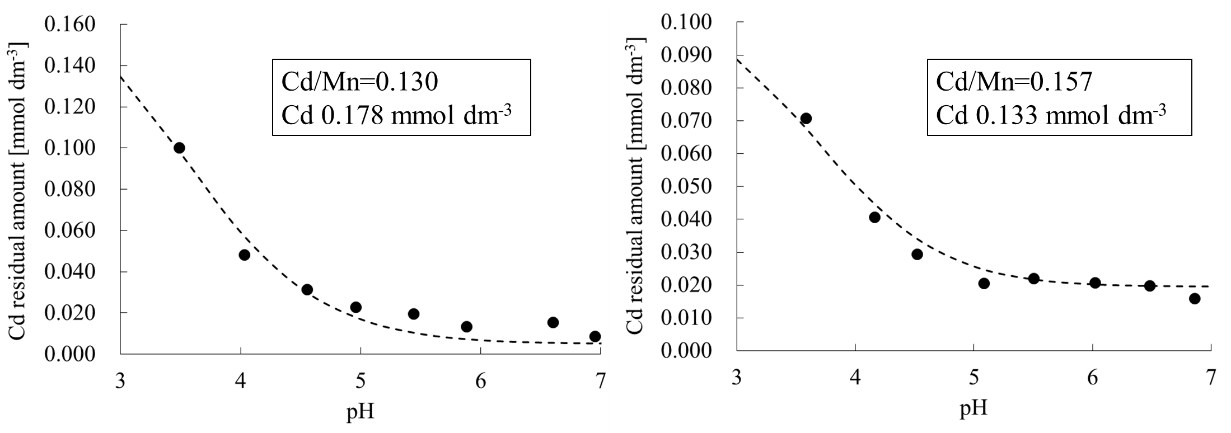
Figure S3**. Cd(II) removed by MnO_2_ adsorption as a function of pH. Black dots are experimental values, and dotted lines are the results of thermodynamic calculations.
